# Supplementary material for: Intracellular energy controls dynamics of stress-induced ribonucleoprotein granules
Source: Nat Commun. 2022 Sep 23;13:5584. doi: 10.1038/s41467-022-33079-1 (PMC9508253; doi:10.1038/s41467-022-33079-1)
Supplement: Supplementary file 1 — Supplementary Information [file 41467_2022_33079_MOESM1_ESM.pdf]

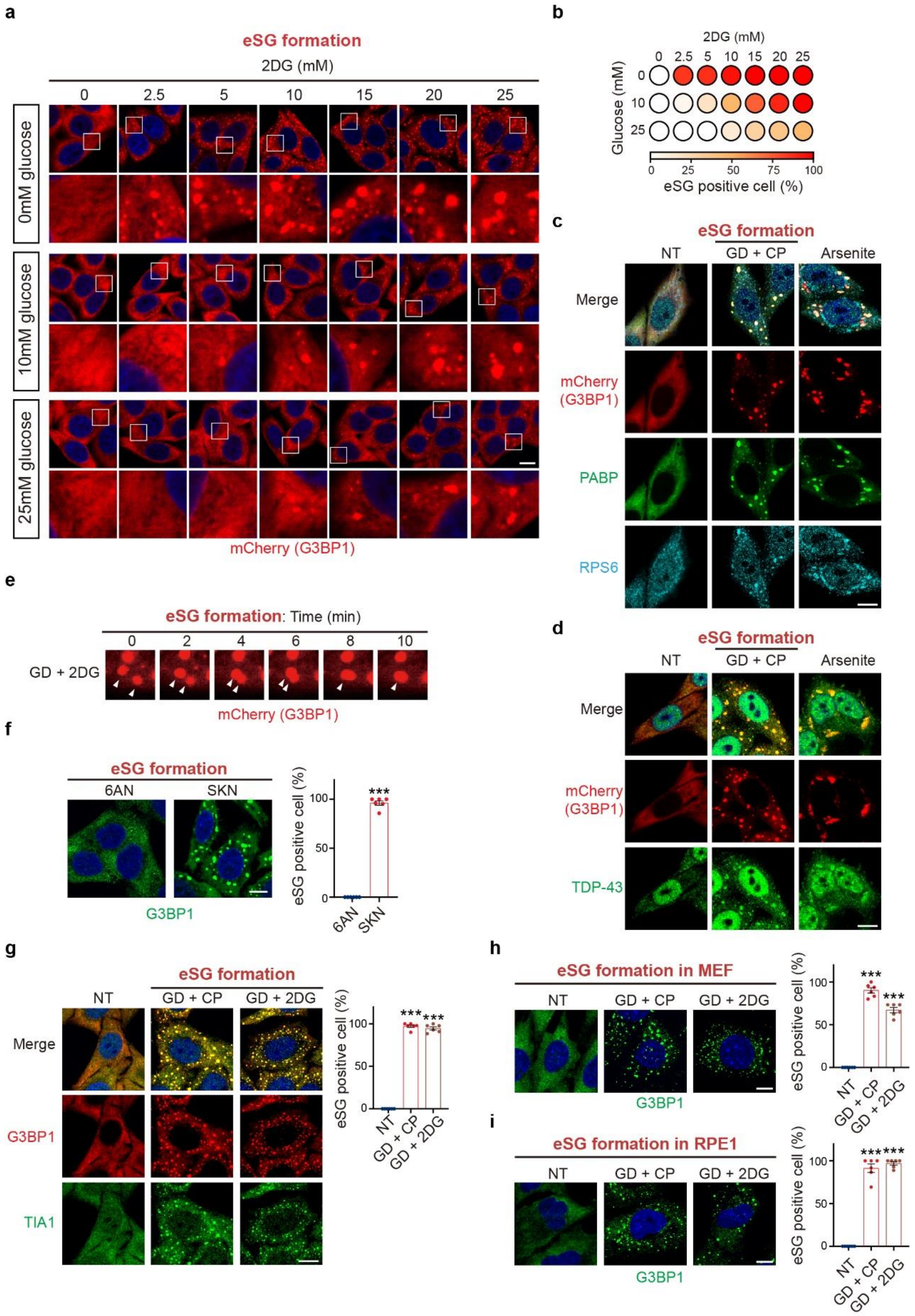

Supplementary Fig. 1. Inhibition of glycolysis, but not PPP, triggers eSG formation.

a,b, Representative images (**a**) and quantifications (**b**) (n = 3) of eSG formation in mCherry-G3BP1-expressing cells treated with increasing concentrations of 2DG along with indicated glucose concentrations for 1 h.

c,d Representative images of SGs or eSGs in HeLa cells stably expressing mCherry-G3BP1 with the indicated

treatments. Cells were immunostained with anti-PABP, RPS6, and TDP-43 antibodies.

e, A representative fusion of eSGs during glycolytic blockage-induced energy deficiency condition in an mCherry-G3BP1-expressing cell is shown.

f,**g**, Representative images and quantification of eSGs formed in HeLa cells treated with 6AN (250 μM) or SKN (25 μM) (**f**) or the indicated energy deficiencies (**g**) for 1 h. The eSGs were revealed by immunofluorescence (IF) of G3BP1 (**f**) or G3BP1 combined with TIA1 (**g**) and assessed as the percentages of eSG positive cells (n = 6).

h,**i**, Images of eSGs formed in MEF (**h**) or human RPE1 cells (**i**) after the indicated treatments for 1 h. eSGs were visualized and quantified by G3BP1 IF (n = 6).

Nuclei were visualized by DAPI staining (blue). Data are shown as means ± SEM, analyzed by unpaired two-sided Student's *t*-test. *** $P < 0.001$. Scale bars, 10 μm.

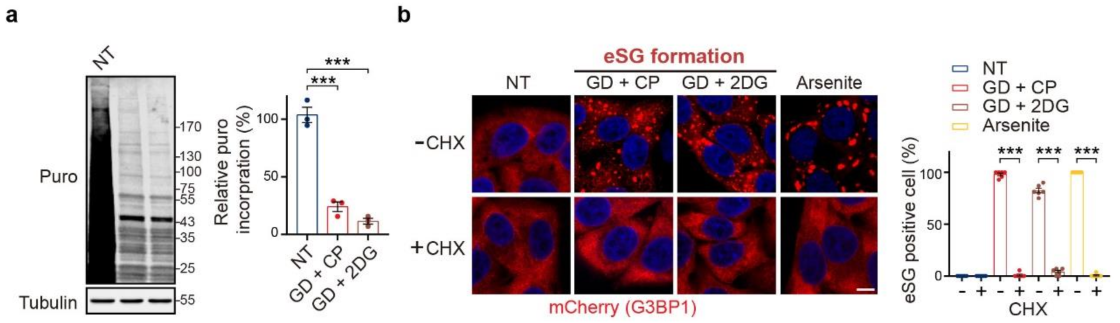

Supplementary Fig. 2. Inhibition of glycolysis causes translational inhibition, and eSG formation is dependent on polysome disassembly.

a, HeLa cells after 1 h of the indicated treatments were subjected to the ribopuromycinylation assay and immunoblotting using an antibody detecting the incorporated puromycin (Puro) to quantify the translational rates (n = 3).

b, HeLa cells stably expressing mCherry-G3BP1 were treated with indicated energy deficiency for 1 h in the presence or absence of cycloheximide (CHX). eSGs were quantified as the percentages of eSG-positive cells (n = 6).

Nuclei were visualized by DAPI staining (blue). Data are shown as means ± SEM, analyzed by unpaired two-sided Student's *t*-test. *** P < 0.001. Scale bars, 10 μm.

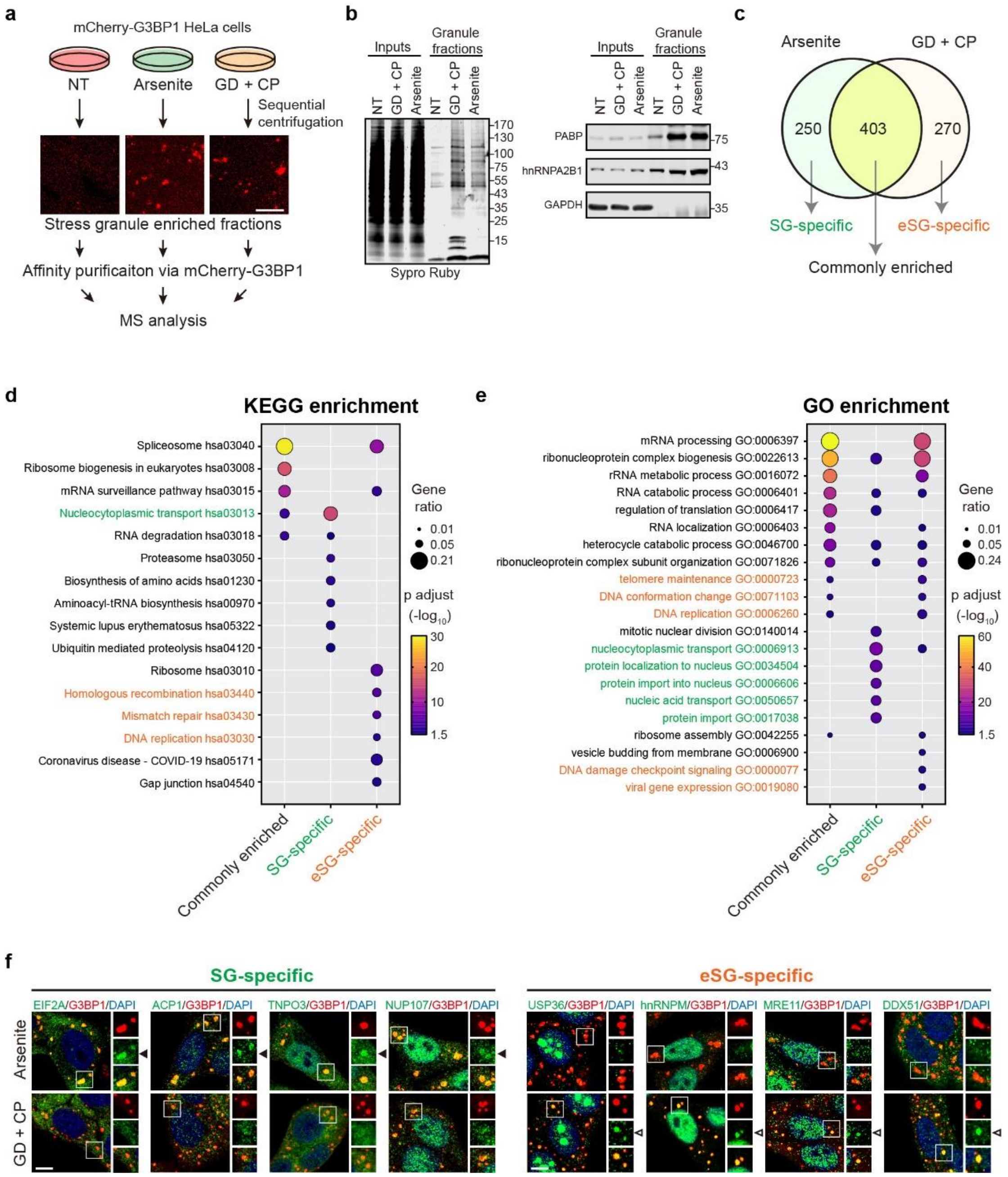

Supplementary Fig. 3. Protein compositions of SGs and eSGs.

a, Scheme for the preparation of stress granule enriched fractions from HeLa cells stably expressing mCherry-G3BP1.

b, Gels stained by Sypro Ruby demonstrating total protein amounts, western blots for two stress granule markers, PABP, hnRNPA2B1, and a non-granule protein, GAPDH, in cell lysates (Inputs) and purified stress granule fractions are shown.

c, Venn diagram depicting the number of the 923 granule proteins identified in either the SG or eSG fraction.

d,e, KEGG (**d**) or GO (**e**) pathway enrichment analysis of the SG- or eSG-enriched proteins and the proteins common to both groups. The terms of the significantly enriched pathways (adjust $p < 0.05$), gene ratio, and the significance of the enrichment (adjust p) are given. The specific SG- or eSG-associated biological processes that were spotted by both KEGG and GO analyses were highlighted in green or orange, respectively.

f, HeLa cells were stressed with arsenite by glycolysis inhibition (GD+ CP) for 1 h to trigger SG or eSG, respectively. Stress granules were visualized by G3BP1 IF, and the granule localization of four top-ranked SG-specific proteins (EIF2A, ACP1, TNPO3, or NUP107) or eSG-specific proteins (USP36, hnRNPM, MRE11, or DDX51) is shown. Closed arrowheads, proteins specifically localized to SGs; open arrowheads, proteins specifically localized to eSGs. Nuclei were visualized by DAPI staining (blue). Scale bars, 10 μm.

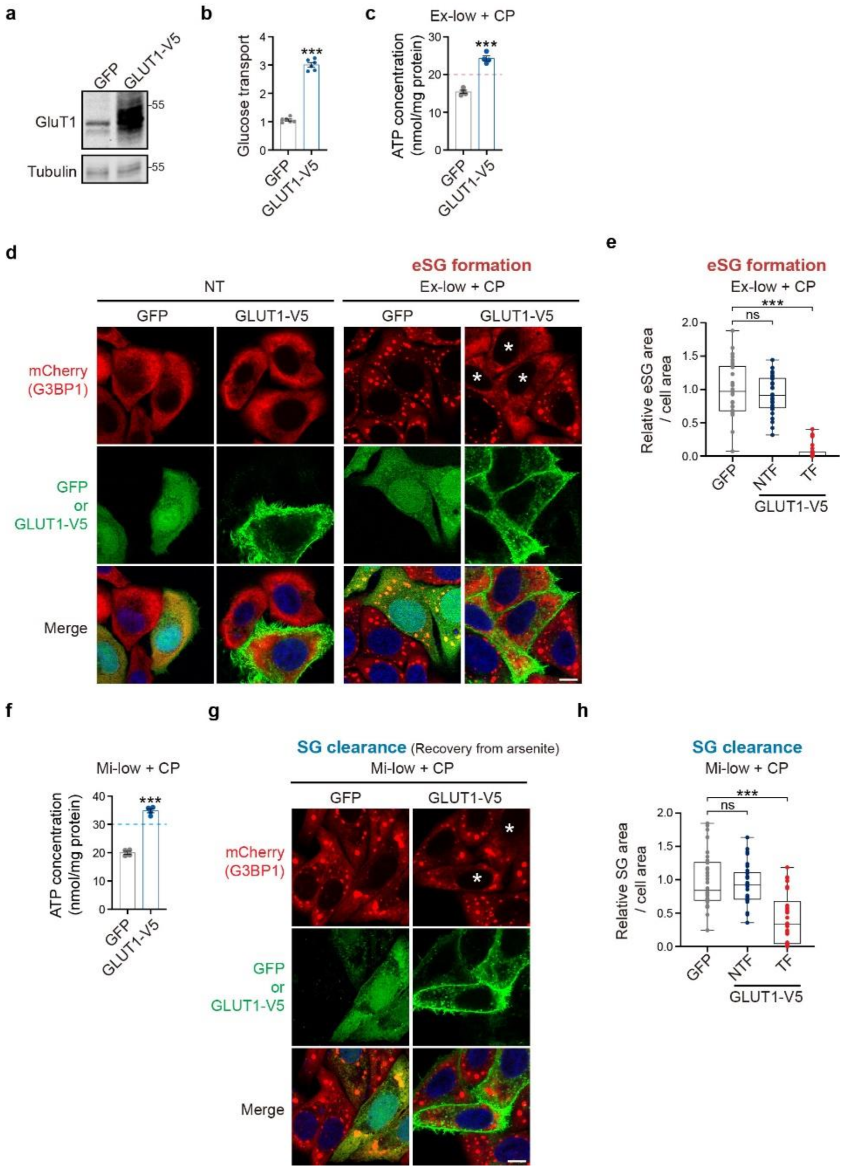

Supplementary Fig. 4. Overexpression of GLUT1 Diminishes eSG formation and restores the SG clearance in cells treated with glycogenolysis inhibition in limited glucose.

a, Western blot analysis of GLUT1 levels in HeLa cells transiently overexpressing either GFP or V5-tagged GLUT1 (GLUT1-V5).

b, Glucose transport assay of HeLa cells overexpressing GFP or GLUT1-V5 (n = 6).

c, Intracellular ATP concentrations in HeLa cells overexpressing GFP or GLUT1-V5 and treated with severe energy deficiency induced by the inhibition of glycogenolysis in physiologically Ex-low glucose (Ex-low + CP) (n = 4). The ATP level below which eSG formation was triggered is indicated by dotted lines.

d, Representative images of the eSGs triggered by the indicated treatments for 1 h in HeLa cells stably expressing mCherry-G3BP1 and transfected with either GFP or GLUT1-V5. Asterisks indicate cells with over-expressed GLUT1-V5 and diminished eSGs.

e, Quantification of (**d**) as the relative eSG area per cell in cells transfected with either GFP (GFP) or GLUT1-V5 (TF) or in non-transfected (NTF) cells (n = 30 cells from three independent experiments).

f, Intracellular ATP concentrations in HeLa cells overexpressing GFP or GLUT1-V5 and treated with moderate energy deficiency introduced by the inhibition of glycogenolysis in physiologically Mi-low glucose (Mi-low + CP) (n = 4). The ATP level below which SG clearance was impaired is indicated by dotted lines.

g, Representative images of the persistent SGs after recovery from arsenite treatment for 1 h in the presence of the indicated energy deficiency in HeLa cells stably expressing mCherry-G3BP1 and transfected with either GFP or GLUT1-V5. Asterisks indicate cells with over-expressed GLUT1-V5 and a reduction in persistent SGs.

h, Quantification of (**g**) as the relative area of persistent SGs per cell in GFP, NTF, or TF GLUT1-V5 cells (n = 30 cells from three independent experiments).

Nuclei were visualized by DAPI staining (blue). Data are shown as means ± SEM, analyzed by unpaired two-sided Student's *t*-test. * P < 0.05; ** P < 0.01; *** P < 0.001; ns, not significant. Scale bars, 10 μm.

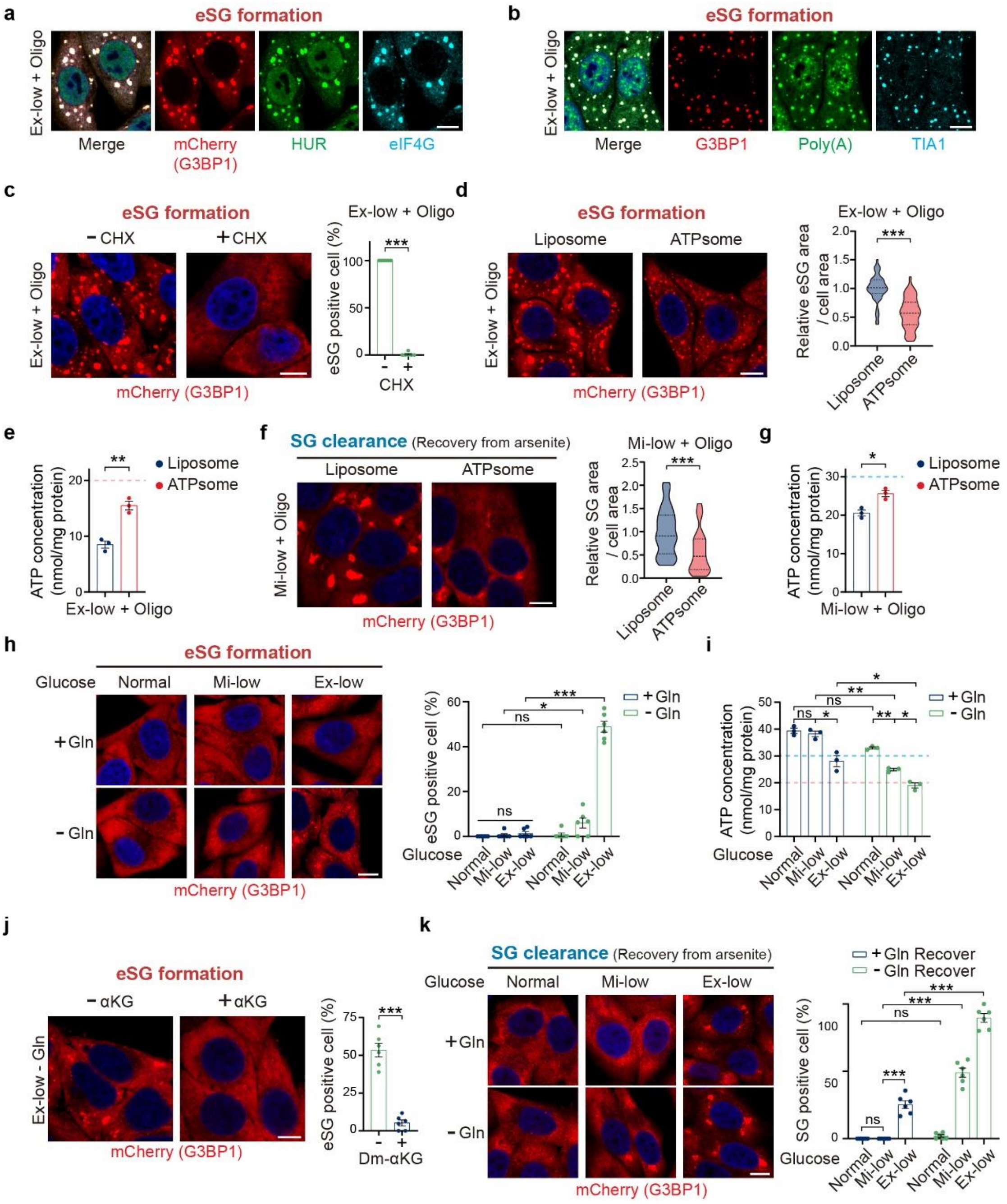

Supplementary Fig. 5. ATP reductions account for the OXPHOS-mediated eSG and SG dynamics.

a,**b**, Representative images of eSGs in HeLa cells, with (**a**) or without (**b**) stable expression of mCherry-G3BP1.

c, HeLa cells stably expressing mCherry-G3BP1 were treated with the indicated energy deficiency in the presence or absence of cycloheximide. The eSGs were quantified as the percentage of eSG-positive cells (n = 6).

d, Representative images and quantification of eSG formation in mCherry-G3BP1-expressing cells in the presence of either control empty liposomes or ATPsome (n = 45 cells).

e, Intracellular ATP concentrations in cells treated as in (**d**) (n = 3). The ATP level below which eSG formation was triggered is indicated by dotted lines.

f, Representative images and quantification of persistent SGs in mCherry-G3BP1-expressing cells after recovery from arsenite treatment. SGs were quantified as the relative eSG areas per cell (n = 45 cells).

g, Intracellular ATP concentrations in cells treated as in (**f**) (n = 3). The ATP level below which SG clearance was impaired is indicated by dotted lines.

h, Images of eSGs in G3BP1-expressing HeLa cells treated with the physiological range of glucose for 1 h in the presence (+Gln) or absence (-Gln) of glutamine. The eSGs were quantified as the percentage of eSG-positive cells (n = 6).

i, Intracellular ATP concentrations in cells treated as in (**h**) (n = 3). The ATP levels below which eSG formation was triggered (pink) or SG clearance was impaired (cyan) are indicated by dotted lines.

j, eSGs formation capacity in G3BP1-expressing HeLa cells in the presence (+αKG) or absence (-αKG) of Dm-αKG. The eSGs were quantified as the percentage of eSG-positive cells (n = 6).

k, Persistent SGs in mCherry-G3BP1 expressing cells after recovery for 1 h after arsenite removal with or without glutamine deprivation. The SGs were quantified as the percentage of SG positive cells (n = 6).

Nuclei were visualized by DAPI staining (blue). Data are shown as means ± SEM, analyzed by unpaired two-sided Student's *t*-test. * $P < 0.05$; ** $P < 0.01$; *** $P < 0.001$; ns, not significant. Scale bars, 10 μm.

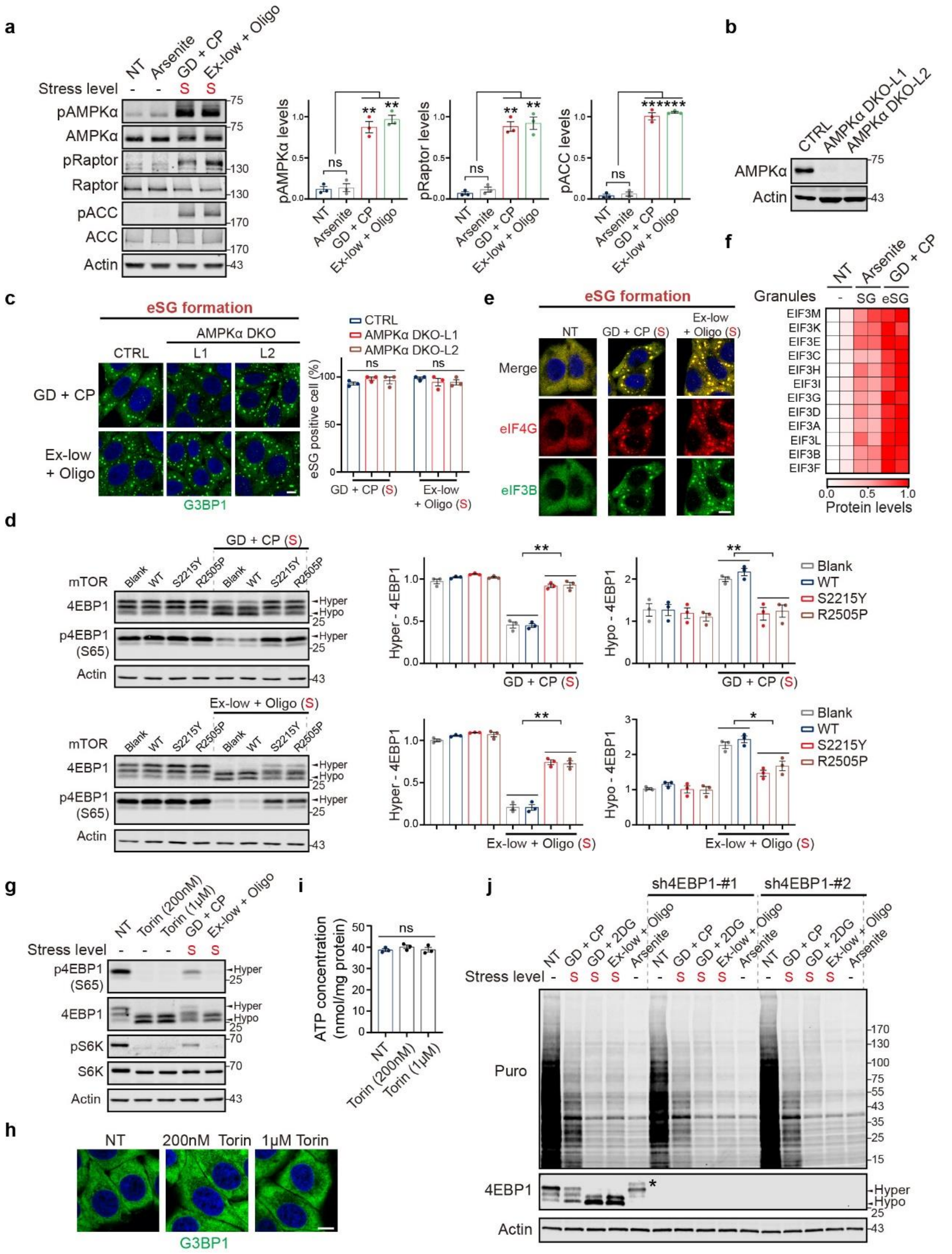

Supplementary Fig. 6. AMPK is not involved in eSG formation, and forced dephosphorylation of 4EBP1 is not sufficient to trigger eSG formation.

a, Western blot analysis of AMPK activity using the indicated antibodies in cells treated with severe energy deficiency or arsenite (n = 3).

b, Western blotting confirms the deletion of AMPKα in two independent HeLa cell lines lacking both the α1 and α2 subunits of AMPK (AMPKα DKO-L1, L2). CTRL: control cells expressing a blank vector.

c, Representative images of eSGs in the control or the two AMPK DKO lines with the indicated treatments. eSGs were quantified as the percentage of eSG-positive cells (n = 3).

d, Cells overexpressing wild-type (WT) or mutant mTOR proteins were subjected to severe energy deficiency. The hyper-4EBP1 levels were assessed by Ser65 phosphorylation, and the hypo-4EBP1 levels were assessed by the relative proportion of the lowest band of 4EBP1 to the total protein (n = 3).

e, Localization of eIF3B in the eSGs marked by eIF4G in cells receiving the indicated treatments.

f, Heat map showing the relative protein levels of 12 eIF3 proteins identified in the purified granule fractions selected from the proteomic analysis (Batch 1) in (Supplementary Table 1).

g, Western blot analysis of the hyper- and hypo-4EBP1 and pS6K levels in HeLa cells treated with Torin1 at the indicated concentrations or with severe energy deficiency for 1 h.

h,**i**, Images of G3BP1 IF and the intracellular ATP concentrations (n = 3) in HeLa cells treated with Torin1 as in (**g**).

j, HeLa cells with or without 4EBP1 depletion were treated with arsenite or the indicated severe energy deficiency for 1 h and then subjected to the ribopuromycinylation assay. The asterisk indicates a band of 4EBP1 that appears after arsenite treatment as a result of unidentified modifications.

Nuclei were visualized by DAPI staining (blue). The stress levels of each treatment are indicated. S, severe energy deficiency. Data are means ± SEM, analyzed by unpaired two-sided Student's *t*-test. * $P < 0.05$; ** $P < 0.01$; *** $P < 0.001$; ns, not significant. Scale bars, 10 μm.

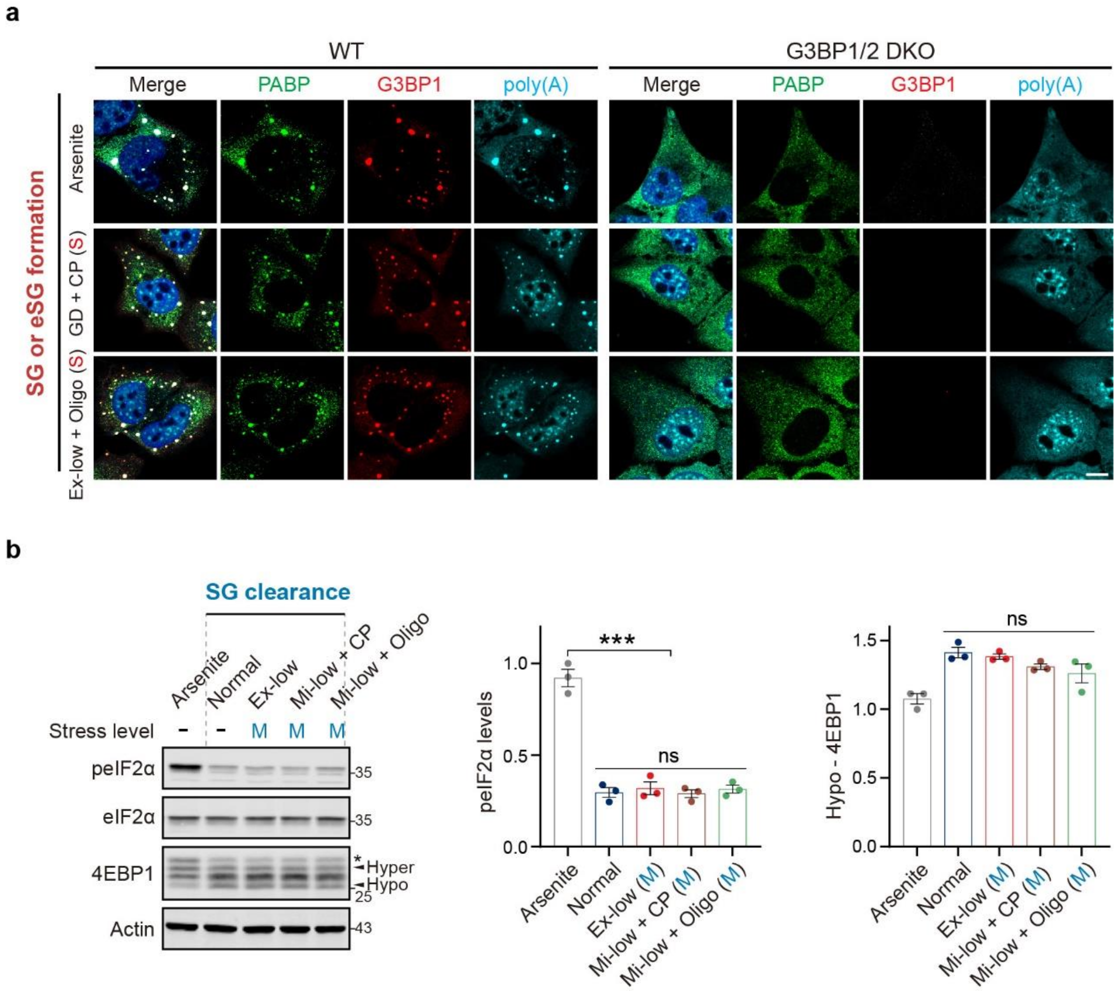

Supplementary Fig. 7. The absence of eSG formation in cells lacking G3BP and unchanged peIF2a and p4EBP1 levels under moderate energy deficiency conditions.

a, Representative images of SGs or eSGs in wild-type or G3BP1/G3BP2 double-knockout (DKO) human U2OS cells. Cells receiving the indicated treatments for 1 h were immunostained with anti-PABP and anti-G3BP1 antibodies, and subjected to mRNA FISH using an oligo-dT20 probe.

b, Western blotting analysis and quantifications of the peIF2a and hypo-4EBP1 levels in cells after the indicated treatments. Hyper- and hypo-4EBP1 are indicated. The asterisk indicates a band of 4EBP1 that appears after arsenite treatment because of some unidentified modification. There was no difference in the eIF2a or 4EBP1 pattern between the cells in normal glucose and the cells with moderate energy deficiency. The stress levels of each treatment are indicated. M, moderate energy deficiency. Data are shown as means ± SEM, analyzed by one-way ANOVA. *** $P < 0.001$; ns, not significant. Scale bars, 10 μm.

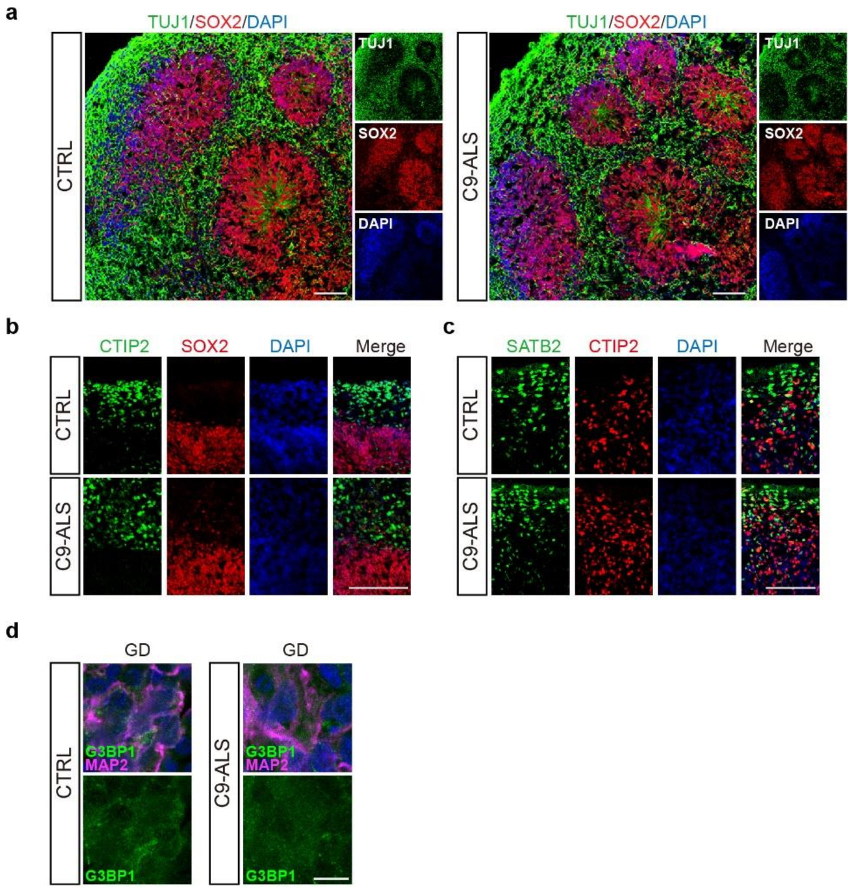

Supplementary Fig. 8. Characterization of cortical organoids from human iPSCs, and lack of eSGs in cortical organoids treated with glucose deprivation.

a, Representative images of the ventricular zone-like structure formed by neurons (TUJ1) and neural progenitor cells (SOX2) in COs from human iPSCs (CTRL 1 and C9-ALS 1) at week 6 of differentiation.

b,**c**, Representative images showing the cortical layer structure when immunostained for CTIP2 (an early development, deep cortical layer marker) and SOX2 at week 8 (b), or for SATB2 (a late development, superficial cortical layer marker) and CTIP2 at week 12 (c).

d, Healthy control COs and C9-ALS COs (100 to 130 days old) treated with glucose deprivation for 1 h were subjected to immunostaining for G3BP1 and a neuronal marker MAP2. Representative images of COs (CTRL 1 and C9-ALS 1) are shown.

Nuclei were visualized by DAPI staining (blue). Scale bars, 100μm (**a**,**b**,**c**), 10 μm (**d**).

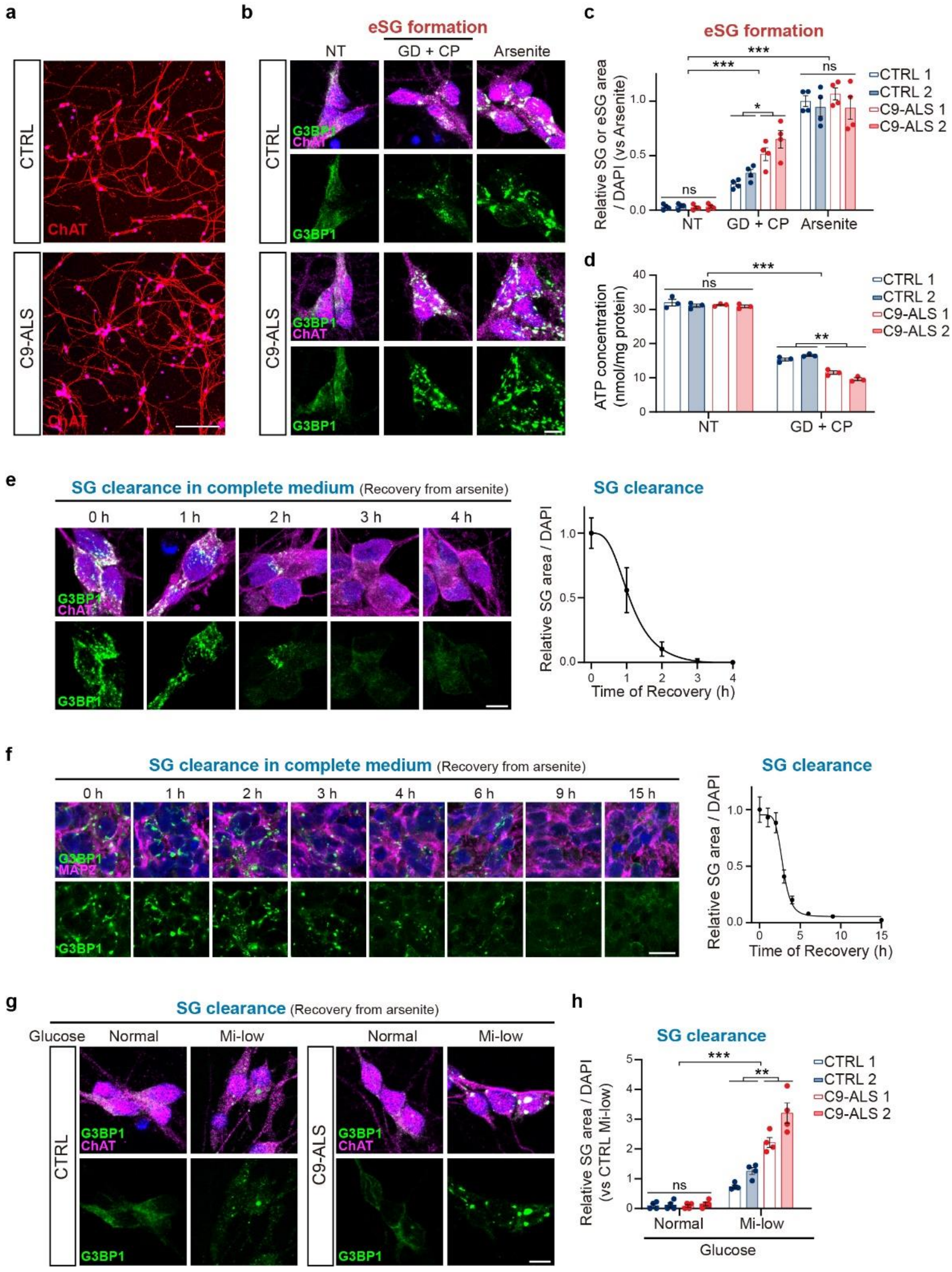

Supplementary Fig. 9. Disrupted eSG formation and SG clearance in C9-ALS patients' motor neurons after energy deficiency, and characterization of SG clearance in COs.

a, Representative images of MNs derived from human iPSCs (CTRL 1 and C9-ALS 1) showing positive for a motor neuron marker, ChAT.

b,**c**, Two healthy control and two C9-ALS motor neurons (MNs) treated with either arsenite or the indicated severe energy deficiency for 1 h were subjected to immunostaining analysis with G3BP1 and ChAT. Representative images of MNs (CTRL 1 and C9-ALS 1) are shown (**b**). The quantification of SGs or eSGs in MNs was assessed by the relative granule area per cell (**c**) (n = 4).

d, Intracellular ATP concentrations in healthy control and C9-ALS MNs with or without the severe energy deficiency treatment as in (**b**) (n = 3).

e, Healthy human MNs treated with arsenite for 1 h to trigger SG formation were allowed to recover in complete medium for the indicated times. The persistent SGs in MNs were visualized by G3BP1 and ChAT IF and quantified as the relative SG areas per cell and normalized to the value at time point 0 (n = 5).

f, Healthy human COs treated with arsenite for 1 h to trigger SG formation were allowed to recover in complete medium for the indicated time. The persistent SGs in CO neurons were visualized by G3BP1 and MAP2 IF and quantified as the relative SG areas per cell, normalized to the value at time point 0 (n = 3).

g,**h**, Healthy control and C9-ALS MNs were allowed to recover from arsenite treatment in mediums with the indicated glucose concentration for 3 h. MNs were subjected to immunostaining analysis with G3BP1 and ChAT IF. Representative images of MNs (CTRL1 and C9-ALS1) are shown (**g**). The quantification of persistent SGs in two healthy control or two C9-ALS MNs is shown in (**h**) (n = 4).

Nuclei were visualized by DAPI staining (blue). Data are means ± SEM, analyzed by unpaired two-sided Student's *t*-test. ** $P < 0.01$; *** $P < 0.001$; ns, not significant. Scale bars, 100μm (**a**), 10 μm (**f**), and 5 μm (**b**,**e**,**g**).
