# Supplementary material for: Intracellular energy controls dynamics of stress-induced ribonucleoprotein granules
Source: Nat Commun. 2022 Sep 23;13:5584. doi: 10.1038/s41467-022-33079-1 (PMC9508253; doi:10.1038/s41467-022-33079-1)
Supplement: Supplementary file 2 — Descriptions for additional Supplementary File [file 41467_2022_33079_MOESM2_ESM.docx]

**Supplementary Data 1. Proteomic analyses of SG and eSG components.**

Description: Protein abundance of the 923 identified granule proteins in the purified granule fractions from HeLa cells treated with Arsenite or glycolysis inhibition (GD + CP) for 1 h. Each group contains four biological replicates obtained from two independently performed proteomic analyses. NT: no treatment. The granule enrichments were assessed by both fold changes (FC) and p values (Student’s t-test).
