# Supplementary material for: Intracellular energy controls dynamics of stress-induced ribonucleoprotein granules
Source: Nat Commun. 2022 Sep 23;13:5584. doi: 10.1038/s41467-022-33079-1 (PMC9508253; doi:10.1038/s41467-022-33079-1)
Supplement: Supplementary file 4 — Reporting Summary [file 41467_2022_33079_MOESM4_ESM.pdf]

nature portfolio

Corresponding author(s): Tao Wang; Jiou Wang

Last updated by author(s): Jul 24, 2022

Reporting Summary

Nature Portfolio wishes to improve the reproducibility of the work that we publish. This form provides structure for consistency and transparency in reporting. For further information on Nature Portfolio policies, see our Editorial Policies and the Editorial Policy Checklist.

Statistics

For all statistical analyses, confirm that the following items are present in the figure legend, table legend, main text, or Methods section.

n/a	Confirmed	
☐	☒	The exact sample size (*n*) for each experimental group/condition, given as a discrete number and unit of measurement
☐	☒	A statement on whether measurements were taken from distinct samples or whether the same sample was measured repeatedly
☐	☒	The statistical test(s) used AND whether they are one- or two-sided<br>*Only common tests should be described solely by name; describe more complex techniques in the Methods section.*
☐	☒	A description of all covariates tested
☐	☒	A description of any assumptions or corrections, such as tests of normality and adjustment for multiple comparisons
☐	☒	A full description of the statistical parameters including central tendency (e.g. means) or other basic estimates (e.g. regression coefficient) AND variation (e.g. standard deviation) or associated estimates of uncertainty (e.g. confidence intervals)
☐	☒	For null hypothesis testing, the test statistic (e.g. *F*, *t*, *r*) with confidence intervals, effect sizes, degrees of freedom and *P* value noted<br>*Give P values as exact values whenever suitable.*
☒	☐	For Bayesian analysis, information on the choice of priors and Markov chain Monte Carlo settings
☒	☐	For hierarchical and complex designs, identification of the appropriate level for tests and full reporting of outcomes
☒	☐	Estimates of effect sizes (e.g. Cohen's *d*, Pearson's *r*), indicating how they were calculated

Our web collection on statistics for biologists contains articles on many of the points above.

Software and code

Policy information about availability of computer code

Data collection	Imaging data were collected using Leica LAS X Software (v3.7.4.23463).
Data analysis	Images were analyzed using Fiji software (ImageJ 1.52p). Data were plotted with GraphPad Prism (v9.3.1). The band intensity in immunoblots was determined using Bio-Rad Quantity One software (v4.6.2). Proteome Discoverer (v2.4.1.15) was used for quantitation and identification of proteins. The list of granule proteins were subjected to the functional annotation analysis by using the Clusterprofiler software (v4.4.4).

For manuscripts utilizing custom algorithms or software that are central to the research but not yet described in published literature, software must be made available to editors and reviewers. We strongly encourage code deposition in a community repository (e.g. GitHub). See the Nature Portfolio guidelines for submitting code & software for further information.

Data

Policy information about availability of data

All manuscripts must include a data availability statement. This statement should provide the following information, where applicable:

- Accession codes, unique identifiers, or web links for publicly available datasets
- A description of any restrictions on data availability
- For clinical datasets or third party data, please ensure that the statement adheres to our policy

Source data are provided with this paper. The mass spectrometry proteomics data have been deposited to the ProteomeXchange Consortium with the accession number PXD035747 [http://proteomecentral.proteomexchange.org/cgi/GetDataset?ID=PXD035747]. All the other data supporting the findings of this study are available within the article and its supplementary materials. This study using iPSCs and human tissue organoids is approved by the Institutional Review Board at the Johns Hopkins University.

Field-specific reporting

Please select the one below that is the best fit for your research. If you are not sure, read the appropriate sections before making your selection.

- [x] Life sciences
- [ ] Behavioural & social sciences
- [ ] Ecological, evolutionary & environmental sciences

For a reference copy of the document with all sections, see nature.com/documents/nr-reporting-summary-flat.pdf

Life sciences study design

All studies must disclose on these points even when the disclosure is negative.

Sample size	No statistical methods were used to predetermine sample size. Three to six independent experiments were conducted for each condition for all experiments. Sample size were determined based on our previously published studies (Cell Metabolism, PMID: 33545050; PNAS, PMID: 30442662) to obtain sufficient statistical significance and reproducibility.
Data exclusions	No data were excluded.
Replication	Each figure describes how many times each experiment had been repeated. All attempts to replicate the experiments were successful.
Randomization	All cells or cortical organoids derived from healthy controls or C9-ALS patients iPSCs were randomly allocated into different groups. Staining images were acquired in random fields of view.
Blinding	The investigators were blinded to group allocation during data collection and the assessment of the outcome. Data analysis was performed and confirmed by multiple investigators.

Reporting for specific materials, systems and methods

We require information from authors about some types of materials, experimental systems and methods used in many studies. Here, indicate whether each material, system or method listed is relevant to your study. If you are not sure if a list item applies to your research, read the appropriate section before selecting a response.

Materials & experimental systems

n/a	Involved in the study	
[ ]	[x]	Antibodies
[ ]	[x]	Eukaryotic cell lines
[x]	[ ]	Palaeontology and archaeology
[x]	[ ]	Animals and other organisms
[ ]	[x]	Human research participants
[x]	[ ]	Clinical data
[x]	[ ]	Dual use research of concern

Methods

n/a	Involved in the study	
[x]	[ ]	ChIP-seq
[x]	[ ]	Flow cytometry
[x]	[ ]	MRI-based neuroimaging

Antibodies

Antibodies used

For IF:
anti-HUR (Santa Cruz Biotechnology, sc-5261, 1:100 dilution), anti-eIF4G (Cell Signaling Technology, 2469, 1:200 dilution), and anti-TDP-43 (Proteintech Group, 10782-2-AP, 1:200 dilution), anti-G3BP1 (BD Bioscience, 611126, 1:800 dilution), anti-V5 tag (Thermo Fisher, 460705, 1:500 dilution), anti-TIA1 (Proteintech Group, 12133-2-AP, 1:200 dilution), anti-PABP (Santa Cruz Biotechnology, sc-32318, 1:100 dilution), anti-EIF2A (Proteintech Group, 11233-1-AP, 1:200 dilution), anti-ACP1 (Proteintech Group, 22214-1-AP, 1:200 dilution), anti-TNPO3 (Thermo Fisher, MA5-34790, 1:200 dilution), anti-NUP107 (Proteintech Group, 19217-1-AP, 1:200 dilution), anti-USP36 (Proteintech Group, 14783-1-AP, 1:200 dilution), anti-hnRNPM (Proteintech Group, 26897-1-AP, 1:200 dilution), anti-MRE11 (Cell Signaling Technology, 4895, 1:200 dilution), anti-DDX51 (Proteintech Group, 20149-1-AP, 1:200 dilution), anti-4EBP1 (Cell Signaling Technology, 9644, 1:200 dilution), anti-eIF3B (Santa Cruz Biotechnology, sc137214, 1:100 dilution), anti-ChAT (Sigma, AB143, 1:400 dilution), anti-TUJ1 (GeneTex, GTX85469, 1:800 dilution), anti-SOX2 (Thermo Fisher, MA1-014, 1:200 dilution), anti-CTIP2 (Cell Signaling Technology, 12120, 1:200 dilution), anti-SATB2 (Sigma, AMAB90682, 1:200 dilution), and anti-MAP2 (Abcam, ab32454, 1:800 dilution).
For immunoblotting:
Anti-puromycin (Sigma, MABE343, 1:4000 dilution); anti-PABP (Santa Cruz Biotechnology, sc-32318, 1:500 dilution); anti-hnRNPA2B1 (Santa Cruz Biotechnology, sc-10036, 1:500 dilution); anti-GLUT1 (Santa Cruz Biotechnology, sc-377228, 1:500 dilution); anti-pAMPKα (Thr172) (Cell Signaling Technology, 50081, 1:1000 dilution); anti-AMPKα (Cell Signaling Technology, 5831, 1:2000 dilution); anti-pRaptor (Ser792) (Cell Signaling Technology, 2083, 1:1000 dilution); anti-Raptor (Cell Signaling Technology, 2280, 1:1000 dilution); anti-pACC (Ser79) (Cell Signaling Technology, 3661, 1:1000 dilution); anti-ACC (Cell Signaling Technology, 3676, 1:1000 dilution); anti-peIF2α (Ser51) (Cell Signaling Technology, 9721, 1:1000 dilution); anti-eIF2α (Cell Signaling Technology, 5324, 1:1000 dilution); anti-eIF4G (Cell Signaling Technology, 2469, 1:3000 dilution); anti-eIF4E (Cell Signaling Technology, 2067, 1:2000 dilution); anti-p4EBP1 (Ser65) (Cell Signaling Technology, 9451, 1:1000 dilution); anti-4EBP1 (Cell Signaling Technology, 9644, 1:2000 dilution); anti-pS6K

(Thr389) (Cell Signaling Technology, 9234, 1:1000 dilution); anti-S6K (Cell Signaling Technology, 9202, 1:2000 dilution); anti-β-tubulin (Cell Signaling Technology, 2128, 1:2000 dilution); and anti-β-actin (Santa Cruz Biotechnology, sc-47778, 1:5000 dilution).

Validation

All antibodies were obtained from companies that reported validation in mammalian cells used in the current study.

For IF:
1. anti-HUR (Santa Cruz Biotechnology, sc-5261)
https://www.scbt.com/p/hur-antibody-3a2
Reactivity: mouse, rat, human and Xenopus.
Application: Western blotting, Immunoprecipitation, Immunofluorescence, Immunohistochemistry, and ELISA.
2. anti-eIF4G (Cell Signaling Technology, 2469)
https://www.cellsignal.com/products/primary-antibodies/eif4g-c45a4-rabbit-mab/2469
Reactivity: human, mouse, rat, Monkey
Application: Western Blotting, Immunohistochemistry, Immunofluorescence, Flow Cytometry
3. anti-TDP-43 (Proteintech Group, 10782-2-AP)
https://www.ptglab.com/products/TARDBP-Antibody-10782-2-AP.htm
Reactivity: Human, Mouse, Rat, Zebrafish, Kak
Application: Western blotting, Immunofluorescence, Immunohistochemistry.
4. anti-G3BP1 (BD Bioscience, 611126)
https://www.bdbiosciences.com/en-us/search-results?searchKey=611126
Reactivity: Human.
Application: Western blotting, Immunofluorescence.
5. anti-V5 tag (Thermo Fisher, 460705)
https://www.thermofisher.com/antibody/product/V5-Tag-Antibody-Monoclonal/R960-25
Reactivity: Tag.
Application: Western blotting, Immunoprecipitation, Immunofluorescence, Immunohistochemistry, ChIP, RNA Immunoprecipitation, Dot blot, in situ PLA, and ELISA.
6. anti-TIA1 (Proteintech Group, 12133-2-AP)
https://www.ptglab.com/products/TIA1-Antibody-12133-2-AP.htm
Reactivity: Human, Mouse, Rat.
Application: Western blotting, Immunofluorescence, Immunohistochemistry, Immunoprecipitation.
7. anti-PABP (Santa Cruz Biotechnology, sc-32318)
https://www.scbt.com/p/pabp-antibody-10e10
Reactivity: human and Xenopus laevis.
Application: Western blotting, Immunoprecipitation, Immunofluorescence, Immunohistochemistry.
8. anti-EIF2A (Proteintech Group, 11233-1-AP)
https://www.ptglab.com/products/EIF2A-Antibody-11233-1-AP.htm
Reactivity: Human, Mouse, Rat.
Application: Western blotting, Immunofluorescence, Immunohistochemistry, Immunoprecipitation.
9. anti-ACP1 (Proteintech Group, 22214-1-AP)
https://www.ptglab.com/products/ACP1-Antibody-22214-1-AP.htm
Reactivity: Human, Mouse, Rat.
Application: Western blotting, Immunofluorescence, Immunohistochemistry.
10. anti-TNPO3 (Thermo Fisher, MA5-34790)
https://www.thermofisher.com/antibody/product/TNPO3-Antibody-clone-JG38-77-Recombinant-Monoclonal/MA5-34790
Reactivity: Human.
Application: Western blotting, Immunofluorescence, Immunohistochemistry.
11. anti-NUP107 (Proteintech Group, 19217-1-AP)
https://www.ptglab.com/products/NUP107-Antibody-19217-1-AP.htm
Reactivity: Human, Mouse, Rat.
Application: Western blotting, Immunoprecipitation, Immunofluorescence.
12. anti-USP36 (Proteintech Group, 14783-1-AP)
https://www.ptglab.com/products/USP36-Antibody-14783-1-AP.htm
Reactivity: Human, Mouse.
Application: Western blotting, Immunoprecipitation, Immunofluorescence, Immunohistochemistry, and ELISA.
13. anti-hnRNPM (Proteintech Group, 26897-1-AP)
Reactivity: Human, Mouse.
Application: Western blotting, Immunofluorescence, and ELISA.
14. anti-MRE11 (Cell Signaling Technology, 4895)
https://www.cellsignal.com/products/primary-antibodies/eif4g-c45a4-rabbit-mab/2469
Reactivity: human, mouse, rat, monkey
Application: Western Blotting, Immunohistochemistry.
15. anti-DDX51 (Proteintech Group, 20149-1-AP)
https://www.ptglab.com/products/DDX51-Antibody-20149-1-AP.htm
Reactivity: Human, Mouse.
Application: Western blotting, Immunoprecipitation, and ELISA.
16. anti-4EBP1 (Cell Signaling Technology, 9644)
https://www.cellsignal.com/products/primary-antibodies/4e-bp1-53h11-rabbit-mab/9644
Reactivity: human, mouse, rat, Monkey

Application: Western Blotting, Immunoprecipitation, Immunohistochemistry, Immunofluorescence, Flow Cytometry
17. anti-eIF3B (Santa Cruz Biotechnology, sc-137214)
https://www.scbt.com/p/eif3eta-antibody-c-5?requestFrom=search
Reactivity: mouse, rat, human.
Application: Western blotting, Immunoprecipitation, Immunofluorescence, Immunohistochemistry, and ELISA.
18. anti-ChAT (Sigma, AB143)
https://www.sigmaaldrich.com/US/en/product/mm/ab143
Reactivity: monkey, mouse, feline, rat, bat, human
Application: Western Blotting, Immunoprecipitation, Immunohistochemistry.
19. anti-TUJ1 (GeneTex, GTX85469)
https://www.genetex.com/Product/Detail/beta-Tubulin-3-Tuj1-antibody/GTX85469
Reactivity: Human, Mouse, Rat.
Application: Western blotting, Immunohistochemistry, Immunofluorescence.
20. anti-SOX2 (Thermo Fisher, MA1-014)
https://www.thermofisher.com/antibody/product/SOX2-Antibody-clone-20G5-Monoclonal/MA1-014
Reactivity: Dog, Fish, Human, Mouse.
Application: Western blotting, Immunoprecipitation, Immunofluorescence, Immunohistochemistry, ChIP, Flow Cytometry.
21. anti-CTIP2 (Cell Signaling Technology, 12120)
https://www.cellsignal.com/products/primary-antibodies/bcl-11b-d6f1-xp-rabbit-mab/12120
Reactivity: Human, Mouse.
Application: Western blotting, Immunoprecipitation, Immunofluorescence, ChIP.
22. anti-SATB2 (Sigma, AMAB90682)
https://www.sigmaaldrich.com/US/en/product/sigma/amab90682
Reactivity: Human.
Application: Immunofluorescence, Immunohistochemistry.
23. anti-MAP2 (Abcam, ab32454)
https://www.abcam.com/map2-antibody-neuronal-marker-ab32454.html
Reactivity: Human, Mouse, Rat.
Application: Western blotting, Immunoprecipitation, Immunohistochemistry, Immunofluorescence, Flow Cytometry.

For immunoblotting:
1. Anti-puromycin (Sigma, MABE343)
https://www.sigmaaldrich.com/US/en/product/mm/mabe343
Reactivity: Human.
Application: Western blotting, Immunofluorescence, Immunohistochemistry.
2. anti-PABP (Santa Cruz Biotechnology, sc-32318)
https://www.scbt.com/p/pabp-antibody-10e10
Reactivity: Human and Xenopus.
Application: Western blotting, Immunoprecipitation, Immunofluorescence, Immunohistochemistry.
3. anti-hnRNPA2B1 (Santa Cruz Biotechnology, sc-10036)
https://www.scbt.com/p/hnrnp-a2-b1-antibody-b-7
Reactivity: mouse, rat, human.
Application: Western blotting, Immunoprecipitation, Immunofluorescence, Immunohistochemistry, and ELISA.
4. anti-GLUT1 (Santa Cruz Biotechnology, sc-377228)
https://www.scbt.com/p/glut1-antibody-a-4?requestFrom=search
Reactivity: Human.
Application: Western blotting, Immunoprecipitation, Immunofluorescence, Immunohistochemistry, and ELISA.
5. anti-pAMPKα(Thr172) (Cell Signaling Technology, 50081)
https://www.cellsignal.com/products/primary-antibodies/phospho-ampka-thr172-d4d6d-rabbit-mab/50081
Reactivity: Human, Mouse, Rat.
Application: Western blotting, Immunoprecipitation, Immunohistochemistry.
6. anti-AMPKα (Cell Signaling Technology, 5831)
https://www.cellsignal.com/products/primary-antibodies/ampka-d5a2-rabbit-mab/5831
Reactivity: Human, Mouse, Rat, Monkey, Bovine.
Application: Western blotting, Immunoprecipitation.
7. anti-pRaptor(Ser792) (Cell Signaling Technology, 2083)
https://www.cellsignal.com/products/primary-antibodies/phospho-raptor-ser792-antibody/2083
Reactivity: Human, Mouse, Rat.
Application: Western blotting.
8. anti-Raptor(Cell Signaling Technology, 2280)
https://www.cellsignal.com/products/primary-antibodies/raptor-24c12-rabbit-mab/2280
Reactivity: Human, Mouse, Rat, Monkey.
Application: Western blotting, Immunoprecipitation.
9. anti-pACC (Ser79) (Cell Signaling Technology, 3661)
https://www.cellsignal.com/products/primary-antibodies/phospho-acetyl-coa-carboxylase-ser79-antibody/3661
Reactivity: Human, Mouse, Rat, Monkey.

Application: Western blotting, Immunoprecipitation, Immunohistochemistry.
10. anti-ACC(Cell Signaling Technology, 3676)
https://www.cellsignal.com/products/primary-antibodies/acetyl-coa-carboxylase-c83b10-rabbit-mab/3676
Reactivity: Human, Mouse, Rat, Hamster.
Application: Western blotting, Immunoprecipitation, Immunohistochemistry, Immunofluorescence, Flow Cytometry.
11. anti-peIF2α (Ser51) (Cell Signaling Technology, 9721)
https://www.cellsignal.com/products/primary-antibodies/phospho-eif2a-ser51-antibody/9721
Reactivity: Human, Mouse, Rat, Monkey, D. melanogaster.
Application: Western blotting.
12. anti-eIF2α (Cell Signaling Technology, 5324)
https://www.cellsignal.com/products/primary-antibodies/eif2a-d7d3-xp-rabbit-mab/5324
Reactivity: Human, Mouse, Rat, Monkey.
Application: Western blotting, Immunoprecipitation, Immunohistochemistry.
13. anti-eIF4G (Cell Signaling Technology, 2469)
https://www.cellsignal.com/products/primary-antibodies/eif4g-c45a4-rabbit-mab/2469
Reactivity: Human, Mouse, Rat, Monkey.
Application: Western blotting, Immunoprecipitation, Immunohistochemistry, Immunofluorescence.
14. anti-eIF4E (Cell Signaling Technology, 2067)
https://www.cellsignal.com/products/primary-antibodies/eif4e-c46h6-rabbit-mab/2067
Reactivity: Human, Mouse, Rat, Monkey.
Application: Western blotting, Immunoprecipitation, Immunohistochemistry.
15. anti-p4EBP1 (Ser65) (Cell Signaling Technology, 9451)
https://www.cellsignal.com/products/primary-antibodies/phospho-4e-bp1-ser65-antibody/9451
Reactivity: Human, Mouse, Rat, Monkey.
Application: Western blotting, Immunoprecipitation.
16. anti-4EBP1 (Cell Signaling Technology, 9644)
https://www.cellsignal.com/products/primary-antibodies/4e-bp1-53h11-rabbit-mab/9644
Reactivity: Human, Mouse, Rat, Monkey.
Application: Western blotting, Immunoprecipitation, Immunohistochemistry, Immunofluorescence.
17. anti-pS6K (Thr389) (Cell Signaling Technology, 9234)
https://www.cellsignal.com/products/primary-antibodies/phospho-p70-s6-kinase-thr389-108d2-rabbit-mab/9234
Reactivity: Human, Mouse, Rat, Monkey.
Application: Western blotting.
18. anti-S6K (Cell Signaling Technology, 9202)
https://www.cellsignal.com/products/primary-antibodies/p70-s6-kinase-antibody/9202
Reactivity: Human, Mouse, Rat, Monkey.
Application: Western blotting, Immunoprecipitation.
19. anti-β-tubulin (Cell Signaling Technology, 2128)
https://www.cellsignal.com/products/primary-antibodies/b-tubulin-9f3-rabbit-mab/2128
Reactivity: Human, Mouse, Rat, Monkey, Zebrafish, Bovine.
Application: Western blotting, Immunohistochemistry, Immunofluorescence.
20. anti-β-actin (Santa Cruz Biotechnology, sc-47778)
https://www.scbt.com/p/beta-actin-antibody-c4?requestFrom=search
Reactivity: Mouse, Rat, Human, Avian, Bovine, Canine, Porcine, Rabbit, Dictyostelium discoideum and Physarum polycephalum.
Application: Western blotting, Immunoprecipitation, Immunofluorescence, Immunohistochemistry, and ELISA.

Eukaryotic cell lines

Policy information about cell lines

Cell line source(s)

HeLa cells (ATCC CCL-2™); MEF cells (homemade, see detail in doi: 10.1371/journal.pgen.1006443; the eIF2α S51A MEF was a kind gift from Dr. Randal J Kaufman, see detail in doi: 10.1016/s1097-2765(01)00265-9.); HEK293 cells (ATCC CRL-1573); RPE1 cells (ATCC CRL-4000); U2OS cell (a kind gift from Dr. J Paul Taylor, see detail in doi: 10.1016/j.cell.2020.03.046.); the iPSCs were obtained from the National Institutes of Health Cell Repositories.

Authentication

G3BP1/G3BP2 DKO U2OS cells and eIF2α S51A MEFs were validated by using immunofluorescence staining of fixed cells and western blot analysis of cell lysates. HeLa, RPE1 and HEK293 cells were originally from ATCC and routinely checked for normal morphology and growth.

Mycoplasma contamination

All cells tested negative from mycoplasma contamination.

Commonly misidentified lines
(See ICLAC register)

None.

Human research participants

Policy information about studies involving human research participants

Population characteristics	This study used two healthy controls and two C9-ALS patients' iPSCs lines obtained from the National Institutes of Health Cell Repositories. Patient IDs: CTRL1, NDS00242; CTRL2, NDS00241; C9-ALS1, NDS00247; C9-ALS2, NDS00239. Detailed information can be found at the NINDS Human Cell and Data Repository (https://stemcells.nindsgenetics.org).
Recruitment	Healthy individuals or C9-ALS patients were recruited by the Target ALS project. No self-selection bias is predicted.
Ethics oversight	This study using iPSCs and human tissue organoids is approved by the Institutional Review Board at the Johns Hopkins University.

Note that full information on the approval of the study protocol must also be provided in the manuscript.
